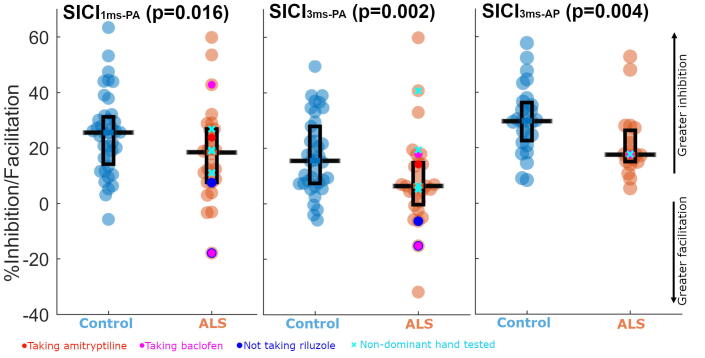# Examining short interval intracortical inhibition with different transcranial magnetic stimulation-induced current directions in ALS

**DOI:** 10.1016/j.cnp.2024.03.001

**Published:** 2024-03-13

**Authors:** Roisin McMackin, Yasmine Tadjine, Antonio Fasano, Matthew Mitchell, Mark Heverin, Friedemann Awiszus, Bahman Nasseroleslami, Richard G. Carson, Orla Hardiman

**Affiliations:** a Discipline of Physiology, School of Medicine, Trinity Biomedical Sciences Institute, Trinity College Dublin, University of Dublin, Ireland; b Academic Unit of Neurology, School of Medicine, Trinity Biomedical Sciences Institute, Trinity College Dublin, University of Dublin, Ireland; c Department of Orthopaedic Surgery, Otto-von-Guericke University, Magdeburg, Germany; d Trinity College Institute of Neuroscience and School of Psychology, Trinity College Dublin, University of Dublin, Ireland; e School of Psychology, Queen’s University Belfast; f Beaumont Hospital, Dublin, Ireland

**Keywords:** Amyotrophic lateral sclerosis, Transcranial magnetic stimulation, Threshold tracking, Short-interval intracortical inhibition, Intracortical facilitation, Coil orientation

## Abstract

**Objective:**

To establish if induced current direction across the motor cortex alters the sensitivity of transcranial magnetic stimulation (TMS)-evoked short-interval intracortical inhibition (SICI) as an ALS biomarker.

**Methods:**

Threshold tracking-TMS was undertaken in 35 people with ALS and 39 controls. Using a coil orientation which induces posterior-anterior (PA)-directed current across the motor cortex, SICI (1 ms and 3 ms interstimulus intervals) and intracortical facilitation (ICF, 10 ms interstimulus interval) were recorded. SICI_3ms_ was also recorded using a coil orientation which induces anterior-posterior (AP)-directed current across the motor cortex.

**Results:**

At group level, SICI_3ms-PA_ (AUROC = 0.7), SICI_3ms-AP_ (AUROC = 0.8) and SICI_1ms_ (AUROC = 0.66) were substantially lower in those with ALS, although there was considerable interindividual heterogeneity. Averaging across interstimulus intervals (ISIs) marginally improved SICI_PA_ sensitivity (AUROC = 0.76). Averaging SICI values across ISIs and orientations into a single SICI measure did not substantially improve sensitivity (AUROC = 0.81) compared to SICI_3ms-AP_ alone. SICI_3ms-AP_ and SICI_3ms-PA_ did not significantly correlate (rho = 0.19, p = 0.313), while SICI_1ms-PA_ and SICI_3ms-PA_ did (rho = 0.37, p = 0.006). Further, those with ALS with the lowest SICI_3ms-PA_ were not those with the lowest SICI_3ms-AP_. ICF was similar between groups (AUROC = 0.50).

**Conclusions:**

SICI_PA_ and SICI_AP_ are uncorrelated measures of motor cortical inhibitory functions which are useful as distinct, unequally affected, measures of disinhibition in ALS.

**Significance:**

Examining both SICI_PA_ and SICI_AP_ may facilitate more comprehensive characterisation of motor cortical disinhibition in ALS.

## Highlights

• SICI measured using posterior-to-anterior induced current direction (SICI_PA_) has been reported to be lower in ALS.
• We find both SICI_PA_ and SICI recorded with anterior-to-posterior induced current direction (SICI_AP_) are lower in ALS.
• SICI_PA_ and SICI_AP_ do not correlate in ALS or controls, indicating that they reflect distinct physiological processes.

## 1 Introduction

While changes in electromyography (EMG) can provide objective biomarkers of lower motor neuron degeneration in ALS (de Carvalho, 2020), there is no universally accepted quantitative biomarker of upper motor neuron dysfunction. Transcranial magnetic stimulation (TMS) is the non-invasive application of a brief magnetic pulse over the scalp which can activate upper motor neurons indirectly, leading to motor unit action potentials, recorded via EMG as motor evoked potentials (MEPs) (McMackin et al., 2019). “Paired pulse” TMS protocols apply a conditioning stimulus (CS) prior to the corticospinal volley-generating test stimulus (TS), with the CS-activated circuitry inducing a net inhibitory or facilitatory influence upon the excitability of upper motor neurons. This leads to a change in the average MEP amplitude evoked by a TS of a specific intensity. As a result, measuring the difference in TS intensity required to evoke a desired MEP amplitude when the CS is present versus absent can be used to quantify motor cortical excitation or inhibition, and the effects of ALS thereon.

Short-interval intracortical inhibition (SICI) refers to a paired pulse protocol in which a subthreshold CS precedes the TS with an interstimulus interval (ISI) between 1 and 7 ms (Fisher et al., 2002, Kujirai et al., 1993, Tankisi et al., 2021a, Vucic and Kiernan, 2006, Ziemann et al., 1996), causing smaller MEPs in response to the TS. SICI is consistently found to be lower in ALS(Sommer et al., 1999, Stefan et al., 2001, Turner et al., 2005, Yokota et al., 1996, Zanette et al., 2002a, Ziemann et al., 1997). Although SICI recorded at different ISIs (e.g. SICI_1ms_ vs SICI_3ms_) are considered to reflect distinct inhibitory processes (SICI_1ms_: tonic GABAergic activity (Stagg et al., 2011) and/or refractory activity (Fisher et al., 2002), SICI_3ms_: GABA-Aergic synaptic activity (Ziemann et al., 2015)), it has been reported that averaging SICI values recorded across a number of ISIs improves discrimination of people with ALS from controls, relative to individual SICI measures alone(Menon et al., 2015, Tankisi et al., 2022). If a longer ISI is used, such as ∼ 10–25 ms, the CS generally increases the amplitude of MEPs evoked by the TS, referred to as intracortical facilitation (ICF) (McMackin et al., 2019). While some studies have reported increased ICF in ALS(Menon et al., 2020, Menon et al., 2018), others have found no change(Agarwal et al., 2021, Stefan et al., 2001, Zanette et al., 2002a, Zanette et al., 2002b, Ziemann et al., 1997).

All studies of SICI in ALS to date have used a posterior-to-anterior (PA) coil orientation. This refers to the induction of electrical currents directed perpendicularly across the precentral gyrus, from posterior to anterior, when the TMS pulse is applied over the motor cortex (Janssen et al., 2015). However, SICI_3ms_ is found to have greater magnitude when an anterior-to-posterior (AP) coil orientation (SICI_AP_) is used(Cerins et al., 2022, Cirillo and Byblow, 2016). It has therefore been proposed that SICI_AP_ could be a more sensitive measure of cortical ALS pathology (Cirillo and Byblow, 2016).

The aims of this study were to determine whether the use of an AP coil orientation could provide additional sensitivity in the detection of ALS pathology compared to the PA orientation, in addition to determining if we could replicate prior reports of sensitive discrimination of people with ALS and controls on the basis of SICI and ICF measures.

## 2 Methods

### 2.1 Ethical approval

Ethical approval was obtained from the ethics committee of St. James's Hospital (REC reference: 2017–02). All participants were over 18 years of age and could provide informed written or verbal consent (in the presence of two witnesses) prior to participation. Except for preregistration, all work was performed in accordance with the Declaration of Helsinki.

### 2.2 Participants

#### 2.2.1 Recruitment

Participants were screened according to the TMS screening questionnaire of Rossi et al. (Rossi et al., 2011) and excluded if contraindications to TMS were identified. Those in the ALS cohort were diagnosed with Possible, Probable or Definite ALS in accordance with the El Escorial Revised Diagnostic Criteria. Healthy controls included neurologically normal individuals recruited from an existing population-based cohort of individuals who registered interest in volunteering for neurological research. A total of 35 people with ALS and 39 healthy controls participated.

### 2.3 Experimental paradigm

#### 2.3.1 Electromyography

Participants were seated upright in a sofa-style chair with wide arm rests and asked to place their arms in their lap or on the arm rest, with the elbow at an angle of approximately 90-120°. EMG activity was recorded at a sampling frequency of 10 kHz from the abductor pollicis brevis (APB) of the dominant hand. For further details see “Electromyography recording protocol” in Supplementary Methods).

#### 2.3.2 Transcranial magnetic stimulation

Monophasic magnetic stimuli were delivered to the scalp via a DuoMag MP Dual stimulator (Deymed Diagnostics s.r.o., Hronov, Czech Republic), equipped with a 50 mm mid-diameter figure-of-eight coil. The axis of intersection between the two loops was oriented at 45° to the sagittal plane to induce PA current flow across the precentral gyrus. Once the scalp hotspot for motor cortex stimulation was identified (see “TMS hotspotting” in Supplementary Methods”), resting motor threshold (RMT, the percentage of maximum stimulator output (%MSO) at which 50 % of stimuli elicit an MEP of at least 50 µV) and threshold hunting target (THT, the %MSO at which 50 % of stimuli elicit an MEP of at least 200 µV) were measured using fully automated parameter estimation by sequential testing (PEST, see “Adaptive threshold tracking” in Supplementary Methods) with PA current flow (PA-RMT and PA-THT). This was followed by rotation of the coil through 180° to achieve AP current flow across M1. The RMT and THT for the AP orientation (AP-RMT and AP-THT) were then obtained at the same position.

Next, paired pulse protocol variants were measured. A 10 ms ISI was used to measure ICF as this ISI was previously reported to provide the greatest ICF-based discrimination between those with ALS and controls (Vucic and Kiernan, 2006), SICI was measured at both 1 and 3 ms ISIs. While PA coil orientation was used for all paired pulse measures of interest, the AP coil orientation also was used to elicit SICI_3ms_. This decision was based on previous studies wherein SICI_3ms_ is found to differ demonstrably when AP and PA orientations are compared, whereas no significant difference was demonstrated for SICI_1ms_ (Cirillo et al., 2018, Cirillo and Byblow, 2016, Mooney et al., 2018, Wessel et al., 2019). The paired pulse protocols investigated were: SICI_1ms-PA_, SICI_3ms-PA_, SICI_3ms-AP_, and ICF_10ms-PA_. For all paired pulse protocols, conditioning pulses were applied at 70 % of the RMT recorded using matching coil orientation. Paired pulse protocols were delivered in random order with rotation of the coil as required between recording of measures employing different orientations. For all TMS protocols, MEP peak-to-peak amplitudes were measured within the 15–50 ms window following test stimulation.

A neuronavigation system was not available for live tracking of coil position relative to target locations. Such navigation systems are very costly and not widely available in research and clinical settings. Further, such tracking systems require participants to wear a head tracker (e.g. a headband or glasses secured to the face) which can be uncomfortable and limit relaxation when worn for the duration of a recording session. We therefore employed a landmark-based system to ensure that pulses were consistently delivered to the same location for both AP and PA orientations (see “Coil positioning protocol” in Supplementary Methods).

#### 2.3.3 Maximum compound muscle action potentials

Maximum compound muscle action potentials (CMAPs) in the dominant hand APB were evoked using electrical stimulation delivered over the median nerve by a Digitimer DS7A stimulator (Digitimer Ltd., Welwyn Garden City, UK) at either the elbow or the wrist (for further information see “Compound muscle action potential measurement” in Supplementary Methods).

### 2.4 Data analysis

#### 2.4.1 Paired pulse TMS analysis

Facilitation/inhibition was defined as the percentage change in test stimulator output necessary to evoke an MEP of target amplitude (200 µV) in the presence of the conditioning stimulus (i.e., the conditioned threshold target, CTT) compared to in its absence (i.e., the THT) as follows:

$$Inhibition/Facilitation\;\left(\%\right)\;=\left(\frac{CTT\;-\;THT}{THT}\times 100\%\;\right)$$

with positive values indicating inhibition and negative values indicating facilitation. To draw comparison to prior studies of “averaged SICI” as an ALS biomarker, mean SICI values were calculated across ISI for PA orientation, and separately across orientations and ISIs, for those individuals for whom all measures could be obtained.

#### 2.4.2 CMAP analysis

For all CMAP-associated signal analyses, latency and maximum peak-to-peak amplitude were examined within the 2–30 ms window following test stimulation. For further details see “Compound muscle action potential analysis” in Supplementary Methods.

#### 2.4.3 Statistics

*Age and sex differences*

Ratio of male to female sex in each cohort was compared by chi-squared test. Ages of each cohort’s participants were compared by Mann-Whitney *U* test.

*CMAP amplitude and latency*

Spearman rank correlation analysis was performed to initially test for the relationship between age and CMAP median latency or maximum amplitude, as groups differed slightly in age distribution. Bonferroni correction was applied, such that differences with p values below 0.025 were deemed significant. As age showed significant correlation with CMAP median latency (rho = 0.42, p = 0.005), CMAP characteristics were compared between groups using linear mixed effects models, with group, site of stimulation, and their interaction, as well as age, incorporated as fixed effects as follows (according to Wilkinson notation):

'Median Latency = Group*Site + Age+(Group*Site|Subject)'
         and
'Maximum Amplitude = Group*Site + Age+(Group*Site|Subject)'

On the basis that Cohen’s d is calculated as group mean difference divided by pooled standard deviation when simply comparing groups (Cohen, 1988), Cohen’s d values for Site [i.e., location of stimulation] and Group were estimated as the coefficient values (corresponding to the mean difference) divided by the standard deviation of the residuals. A 95 % confidence interval for this value was calculated within the fitlme function of MATLAB R2016a.

*Conditioning effects and group differences*

Shapiro-Wilk (Shapiro and Wilk, 1965) and Anderson-Darling (Anderson and Darling, 1952) tests indicated that some TMS measures did not conform to a normal distribution, therefore, non-parametric statistics were employed. To test if the expected SICI/ICF effects were reliably evoked, sign rank testing of control inhibition/facilitation (%) values (compared to zero) was performed for each paired pulse protocol. To account for multiple comparisons, these four p values were corrected to obtain a false discovery rate (FDR) below 5 %, determined by the Benjamini-Hochberg method (Benjamini, 2010), and only those corrected p values below 0.05 were considered significant. To test for abnormalities in SICI/ICF in ALS, Mann-Whitney U testing was performed, comparing inhibition/facilitation values between those with ALS and controls for each single or paired pulse protocol. To account for multiple comparisons, p values were corrected to obtain a positive FDR below 5 % by the Benjamini-Hochberg method (Benjamini, 2010), and only corrected p values below 0.05 were considered significant. Brown-Forsythe testing (Brown and Forsythe, 1974), which is robust to non-normality, was performed for all TMS measures and in the case of all measures a p > 0.05 indicates that the null hypothesis of equal variance between cohorts could not be rejected.

*Potential ceiling effect bias*

In some participants, a ceiling effect occurred where it was not possible to apply sufficiently high stimulus intensities (i.e., >100 % MSO) to counteract an inhibitory conditioning effect. In these participants, some SICI measures could not be measured directly. Inability to record SICI in these individuals could potentially introduce bias to comparative analyses of people with ALS and controls by excluding or underestimating SICI values of those with high thresholds. In most of these participants, MEPs > 50 µV but < 200 µV were repeatedly evoked at 100 % MSO during application of SICI protocols. Further, the difference between these individuals’ threshold stimulation intensities for 200 µV and 50 µV target amplitudes was known in the absence of inhibitory conditioning (i.e., THT minus RMT). We therefore approximated SICI values in these individuals based on the assumption that the difference between the threshold stimulation intensities required to evoke 50 µV and 200 µV does not change with the introduction of the inhibitory conditioning stimulus. Under this assumption, we approximated the true conditioned threshold in these individuals as:

CTT=100%MSO+(THT-RMT)

and used this CTT value to estimate %Inhibition/Facilitation (as per formula above) in these participants. Our group comparison statistical analyses were then repeated, including these approximate values for most of those who were excluded from the primary analysis. This additional, supplementary analysis was performed purely to investigate if the apparent ALS-related abnormalities observed in this study could be driven purely by bias caused by this ceiling effect. However, the assumption upon which we have based these SICI estimates has not yet been proven, and cannot be tested using on this dataset due to lack of SICI measures tracked with a target amplitude of 50 µV. Therefore, we do not recommend the use of this SICI approximation method beyond this purpose.

*Discrimination ability and effect size*

To quantify the potential of paired pulse TMS measures to discriminate people with ALS from controls, area under the receiver operating characteristic curve (AUROC) was calculated for each TMS parameter for each ISI and coil orientation used. Effect size was measured by Hedge’s g, a measure similar to Cohen’s d which includes a correction factor for potential bias caused by smaller sample sizes (Lin and Aloe, 2021). Values and confidence intervals for Hedge’s g and AUROC values were calculated via the “Measures of Effect Size” MATLAB toolbox of Hentschke and Stüttgen (Hentschke and Stüttgen, 2011) applying bootstrapping 1000 times.

*Correlation analyses*

Spearman’s rank correlation analysis was performed to determine the linear association between each TMS measure and 1) disease duration (time since self-reported first symptom onset) and 2) revised ALS functional rating scale (ALSFRS-R) total score. ALSFRS-R score recorded within two months of study participation was available for 26 people with ALS and scores for an additional 4 people with ALS were estimated based on interpolation of two ALSFRS-R measures taken before and after participation. To account for multiple comparisons, p values were corrected (across p values for each TMS measure, separately for ALSFRS-R scores and disease duration) to obtain a positive FDR below 5 % by the Benjamini-Hochberg method (Benjamini, 2010), and only corrected p values below 0.05 were considered significant. Spearman’s rank correlation analysis was also used to investigate the relationship between the different paired pulse TMS measures.

## 3 Results

### 3.1 Final cohort

The minimum requirement for the inclusion of an individual in this analysis was a measurable RMT_PA_, obtained below 100 % MSO. Thirty-five people with ALS (11 female, age median [range]: 64 [41–79] years, 2 left-handed) and thirty-nine controls (12 female, age median [range]: 59 [34–76] years, 1 left-handed) satisfied this requirement (Table 1). The two cohorts were sex- (chi^2^ = 0.0037, p = 0.951) but not age- (p = 0.041) matched. Spearman correlation analyses failed to reveal associations between age and any of the TMS-based measures. Maximum CMAP data were collected from 29 people with ALS and 31 controls who provided RMT_PA_ data. The numbers of datapoints successfully collected for each measurement are given in Table 2.

**Table 1:** **Clinical characteristics of final cohort of 35 people with ALS.** U-Upper limb, L-Lower limb, l-left side, r-right side, e-both sides affected. ALSFRS-R scores measured within 90 days of TMS recording were available for 30 out of 35 people with ALS. Record of El Escorial category at time of diagnosis was retrievable for 25 out of 35 people with ALS.

Time since participant-estimated first symptom onset in months median [range]	19 [7–96]
Time since diagnosis median [range] in months	7 [1–80]
ALSFRS-R total score median [range] for n = 30	41 [17–47]
Region of onset (Spinal/Bulbar/Respiratory)	29/4/2
Site/side of first symptom onset for spinal onset (Ul/Ll/Ur/Lr/Ue/Le)	3/4/6/9/4/3
El Escorial Criterion (Possible/Probable/Definite/Lab Supported Probable)	5/11/7/2

**Table 2:** **Summary statistics for each TMS parameter recorded.** Numbers of ALS and control datasets recorded are listed under Pn and Cn respectively. Group p (g) column values refer to p-value and Hedge’s g for the comparison of ALS and control values. Bolded values represent those correlations deemed significant at a 5 % FDR. 95 % CI – 95 % confidence interval. Numbers of ALS and control datasets excluded due to CTT > 100 % MSO are listed under P > 100 and C > 100 respectively. P values listed are uncorrected. Mean – Mean inhibition values across 1 and 3 ms ISIs. Those coefficient values with corrected p values (at 5 % false discovery rate) < 0.05 are emboldened. RMT – Resting motor threshold. THT – Threshold hunting target. SICI – Short-interval intracortical inhibition. ICF – Intracortical facilitation. ISI – Interstimulus interval. PA – Posterior-to-anterior. AP – Anterior-to-posterior. AUROC – Area under the receiver operating characteristic curve. ^#^/^##^Data for this measure not recorded from one participant/two participants respectively in this cohort due to difficulty relaxing/ time constraints.

Parameter	Orientation	ISI	Cn	C >100	Pn	P >100	Group p (g [95 % CI])	AUROC (95 % CI)
RMT	PA	N/A	39	0	35	0	**0.006 (0.66** **[0.20**–**1.14])**	**0.69** **(0.56**–**0.80)**
	AP	N/A	35	4	25	10	0.610 (0.22 [-0.27–0.75])	0.54 (0.31–0.61)
THT	PA	N/A	39	0	27	8	0.151 (0.22 [-0.24–0.74])	0.60 (0.47–0.74)
	AP	N/A	31	4	21	4	0.136 (0.46 [-0.05–1.08])	0.62 (0.46–0.78)
SICI	PA	1 ms	34	5	24^#^	2	0.035 (-0.61 [-1.14 - −0.09])	0.66 (0.53–0.80)
		3 ms	34	5	26	1	**0.009 (-0.65** **[-1.18- −0.17])**	**0.70** **(0.57**–**0.83)**
		Mean	31	N/A	24	N/A	**0.001 (-0.93** **[-1.57- −0.54])**	**0.76** **(0.63**–**0.88)**
	AP	3 ms	18^#^	12	11^#^	9	**0.008 (-0.93** **[-2.18- −0.14])**	**0.80** **(0.61**–**0.97)**
	AP and PA	3 ms	18	N/A	11	N/A	**0.004 (-1.14** **[-2.28 - −0.33])**	**0.83** **(0.56**–**0.96)**
		Mean	18	N/A	9	N/A	**0.009 (-1.20** **[-2.32 - −0.43])**	**0.81** **(0.68**–**0.95)**
ICF	PA	10 ms	39	0	25^##^	0	1 (0.049 [-0.50–0.51])	0.50 (0.35–0.65)

### 3.2 Differences in TMS measures between people with ALS and controls

#### 3.2.1 Motor thresholds

**Fig. 1:**
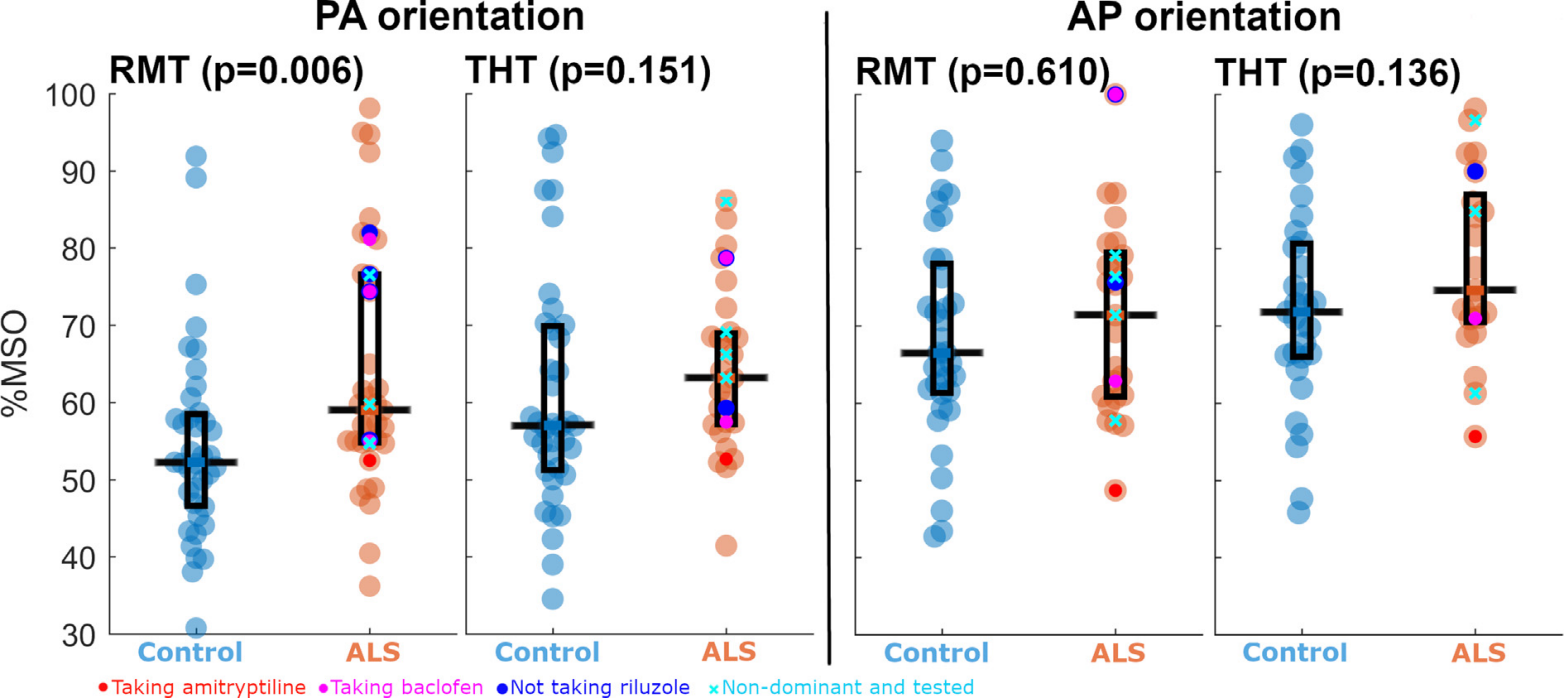
**People with ALS show greater RMT, when PA orientation is used, compared to controls.** Boxes represent the interquartile range, with horizontal black line within each box representing the median. Within the ALS cohort, cyan crosses represent values of those where the non-dominant hand was tested, magenta dots represent values of those taking baclofen, bright red dots represent values of those taking amitriptyline and bright blue dots represent values of those not taking riluzole. P values in subfigure titles are those for Mann-Whitney *U* test comparisons between cohorts. RMT – Resting motor threshold. THT – Threshold hunting target. PA – Posterior-to-anterior induced current direction. AP – Anterior-to-posterior induced current direction. %MSO – Percentage of maximum stimulator output. (For interpretation of the references to colour in this figure legend, the reader is referred to the web version of this article.)

When the PA orientation was used, the 50 µV RMTs of the ALS group were higher (in terms of %MSO) than those of the controls (Fig. 1, Table 2).

#### 3.2.2 SICI

**Fig. 2:**
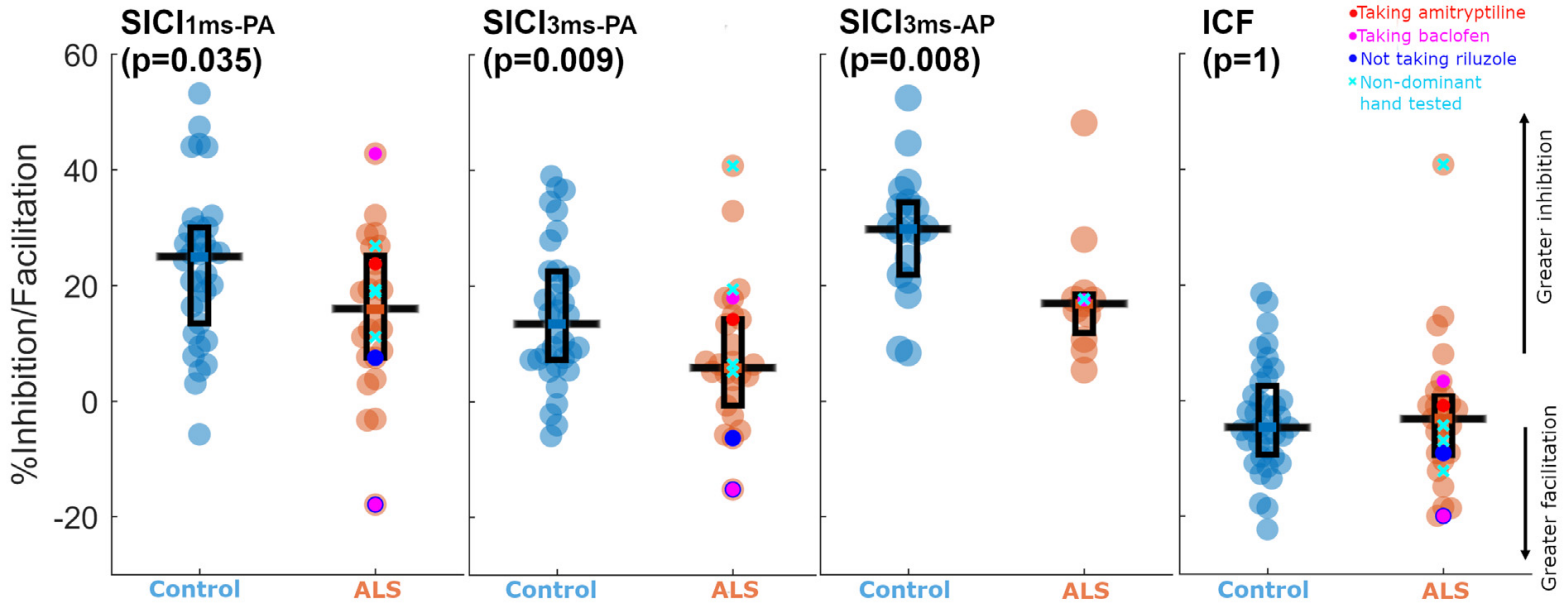
**People with ALS show less SICI when an ISI of 3 ms is used.** Boxes represent the interquartile range, with horizontal black line within each box representing the median. Within the ALS cohort, cyan crosses represent values of those where the non-dominant hand was tested, magenta dots represent values of those taking baclofen, bright red dots represent values of those taking amitriptyline and bright blue dots represent values of those not taking riluzole. P values in subfigure titles are those for Mann-Whitney *U* test comparisons between cohorts. SICI – Short-interval intracortical inhibition. ICF – Intracortical facilitation. PA – Posterior-to-anterior induced current direction. AP – Anterior-to-posterior induced current direction. (For interpretation of the references to colour in this figure legend, the reader is referred to the web version of this article.)

Sign rank testing demonstrated that significant inhibition was achieved in controls (SICI_1ms_ z = 5.03, p < 0.001; SICI_3ms-PA_ z = 4.85, p < 0.001; SICI_3ms-AP_ z = 3.73, p < 0.001) for all SICI measures (Fig. 2). Sign rank testing of paired SICI_3ms-PA_ and SICI_3ms-AP_ values in controls (n = 18) identified that SICI was of significantly greater magnitude when AP coil orientation was employed (p < 0.001). When SICI measures were compared between those with ALS and controls (Table 2), less inhibition was observed in those with ALS in the case of the SICI_3ms-AP_ (Hedge’s g = -0.93) and SICI_3ms-PA_ (Hedge’s g = -0.65) conditions. In respect of the SICI_1ms-PA_, the magnitude of the difference was smaller (Hedge’s g = -0.61) and did not satisfy the FDR adjusted criterion for statistical significance. Inclusion of estimated values for those with conditioned thresholds > 100 % MSO (Table S1), increasing the number of datasets compared, did not change our findings, with the exception that the inhibition arising from SICI_1ms-PA_ was deemed significantly lower in those with ALS than in controls (Figure S2, Table S1). When those taking baclofen or amitriptyline, or those for whom recordings were obtained from the non-dominant hand, were excluded from the analysis of each SICI protocol, the pattern of outcomes did not change for SICI_3ms_ measures (there were no changes to which comparisons were deemed significant at a 5 % FDR).

### 3.3 Discrimination based on combinations of SICI measures

The discrimination of ALS status on the basis of SICI_3ms_ induced inhibition was marginally, although not consistently, greater for AP orientation (AUROC [95 % CI] = 0.80[0.61–0.97]) than for PA orientation (AUROC [95 %CI] = 0.70[0.57–0.83]). Averaging of SICI_PA_ values (when both PA SICI_1ms-PA_ and PA SICI_3ms-PA_ could be quantified in the same individual) increased the effect sizes for the between group comparison (from −0.61 and −0.65 to −0.93), and the resulting AUROC value (AUROC [95 % CI] = 0.76[0.63–0.88]) was similar that registered for SICI_3ms-AP_ alone (see Table 2). The AUROC value was also similar to that obtained for the SICI_3ms-AP_ measure alone for average SICI metrics generated from the mean of SICI_3ms-PA_ and SICI_3ms-AP_ (AUROC = 0.83), or from the mean of SICI_1ms-PA,_ SICI_3ms-PA_ and SICI_3ms-AP_ (AUROC = 0.81).

### 3.4 Relationship between SICI and ICF measures

**Fig. 3:**
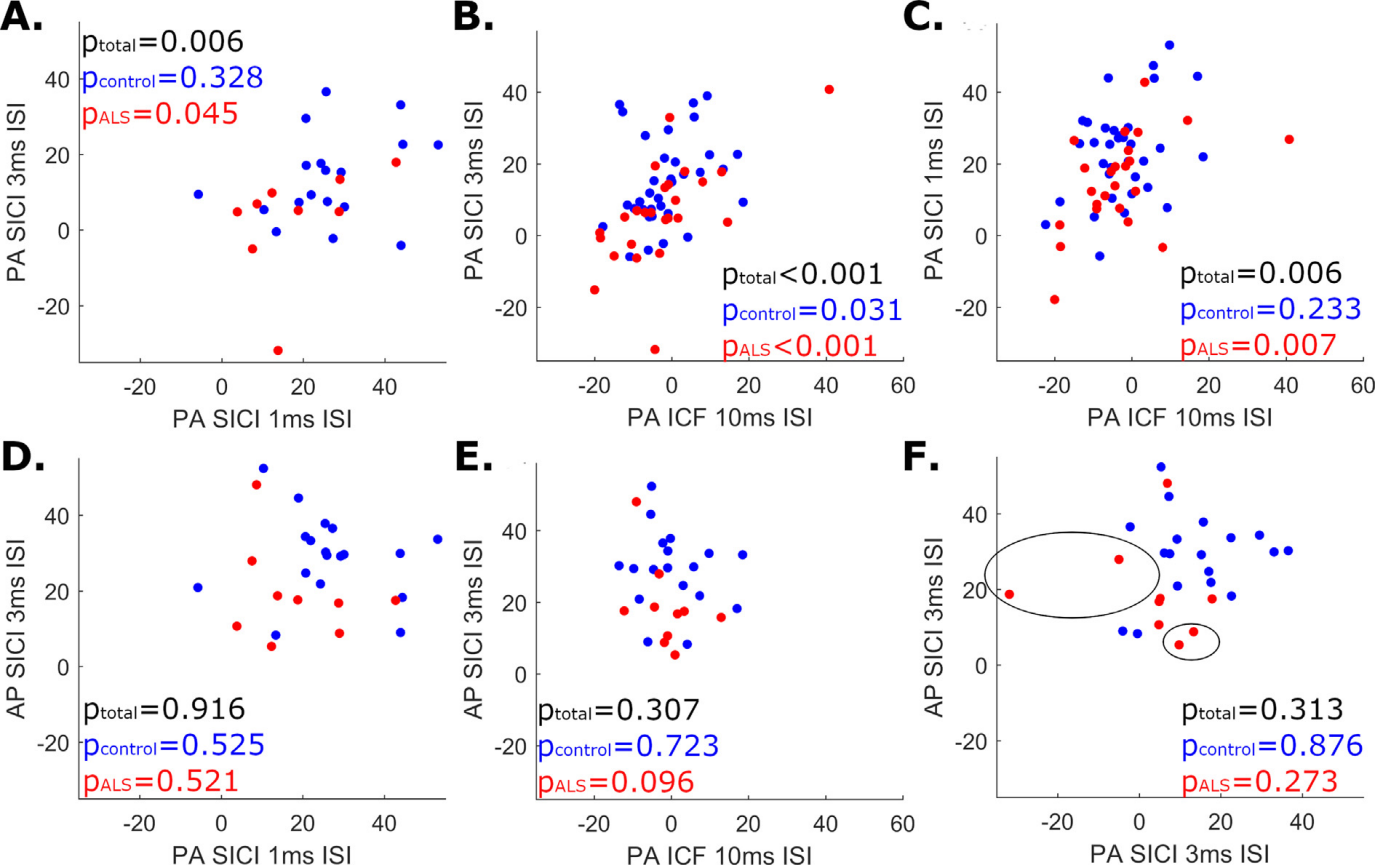
**Scatter plot illustrating the relationships between SICI and ICF values within people with ALS (red) and controls (blue).** Circled values in (F) highlight that the two individuals with ALS with the least SICI_3ms-AP_ had SICI_3ms-PA_ values amongst the highest of the ALS cohort, and vice versa. Spearman’s rank correlation p-values are given for controls only (p_control_), people with ALS only (p_ALS_) and the overall dataset (p_total_). SICI – Short-interval intracortical inhibition. ICF – Intracortical facilitation. PA – Posterior-to-anterior induced current direction. AP – Anterior-to-posterior induced current direction. (For interpretation of the references to colour in this figure legend, the reader is referred to the web version of this article.)

Across participants, significant positive correlations were observed between SICI_1ms-PA_ and SICI_3ms-PA_, as well as between (PA) ICF and SICI_1ms-PA_ and between (PA) ICF and PA SICI_3ms_. By contrast, no significant correlations were identified between SICI_3ms-AP_ and SICI_3ms-PA_, SICI_1ms-PA_ or ICF (Table 3). In keeping with the absence of positive correlation between SICI_PA_ and SICI_3ms-AP_ measures in people with ALS (rho = -0.36, p = 0.273), individuals with ALS displaying the least SICI_3ms-PA_ displayed relatively high SICI_3ms-AP_ values compared to the rest of the ALS cohort and vice versa (Fig. 3F, circled data points).

**Table 3:** **Correlation statistics between SICI and ICF measures across cohorts**. n – Cohort size (individuals for whom both values could be measured), 95% CI – Bootstrapped 95% confidence interval. Bolded values represent those correlations deemed significant at a 5% FDR. SICI – Short-interval intracortical inhibition. ICF – Intracortical facilitation. PA – Posterior-to-anterior orientation. AP – Anterior-to-posterior orientation.

Pair of measures	Controls			ALS			Total group		
	n	rho [95 %CI]	p	n	rho [95 %CI]	p	n	rho [95 %CI]	p
SICI_1ms-PA_ SICI_3ms-PA_	31	0.18 [-0.24–0.53]	0.328	24	0.42 [0.09–0.68]	0.045	**55**	**0.37** **[0.15–0.61]**	**0.006**
SICI_1ms-PA_ ICF	34	0.21 [-0.21–0.54]	0.233	**24**	**0.54** **[0.08–0.85]**	**0.007**	**58**	**0.36** **[0.07–0.58]**	**0.006**
SICI_3ms-PA_ ICF	34	0.37 [-0.11–0.60]	0.031	**25**	**0.64** **[0.32–0.90]**	**<0.001**	**59**	**0.50** **[0.22–0.67]**	**<0.001**
SICI_3ms-AP_ ICF	18	−0.90 [-0.52–0.27]	0.723	10	−0.56 [-0.90–0.01]	0.096	28	−0.20 [-0.43–0.17]	0.307
SICI_1ms-PA_ SICI_3ms-AP_	18	−0.16 [-0.72–0.36]	0.525	11	−0.25 [-0.74–0.62]	0.521	29	−0.021 [-0.43–0.37]	0.916
SICI_3ms-PA_ SICI_3ms-AP_	18	0.04 [-0.38–0.68]	0.876	11	−0.36 [-0.50–0.41]	0.273	29	0.19 [-0.12–0.40]	0.313

### 3.5 Clinical correlations

A negative correlation was observed between ALSFRS-R 5a (cutting food and handling utensils) and THT recorded with PA orientation (rho = -0.64, p < 0.001). No significant correlations between TMS-based measures and ALSFRS-R total scores, other ALSFRS-R subscores, or self-reported disease duration were identified.

### 3.6 Differences in CMAP measures between people with ALS and controls

**Fig. 4:**
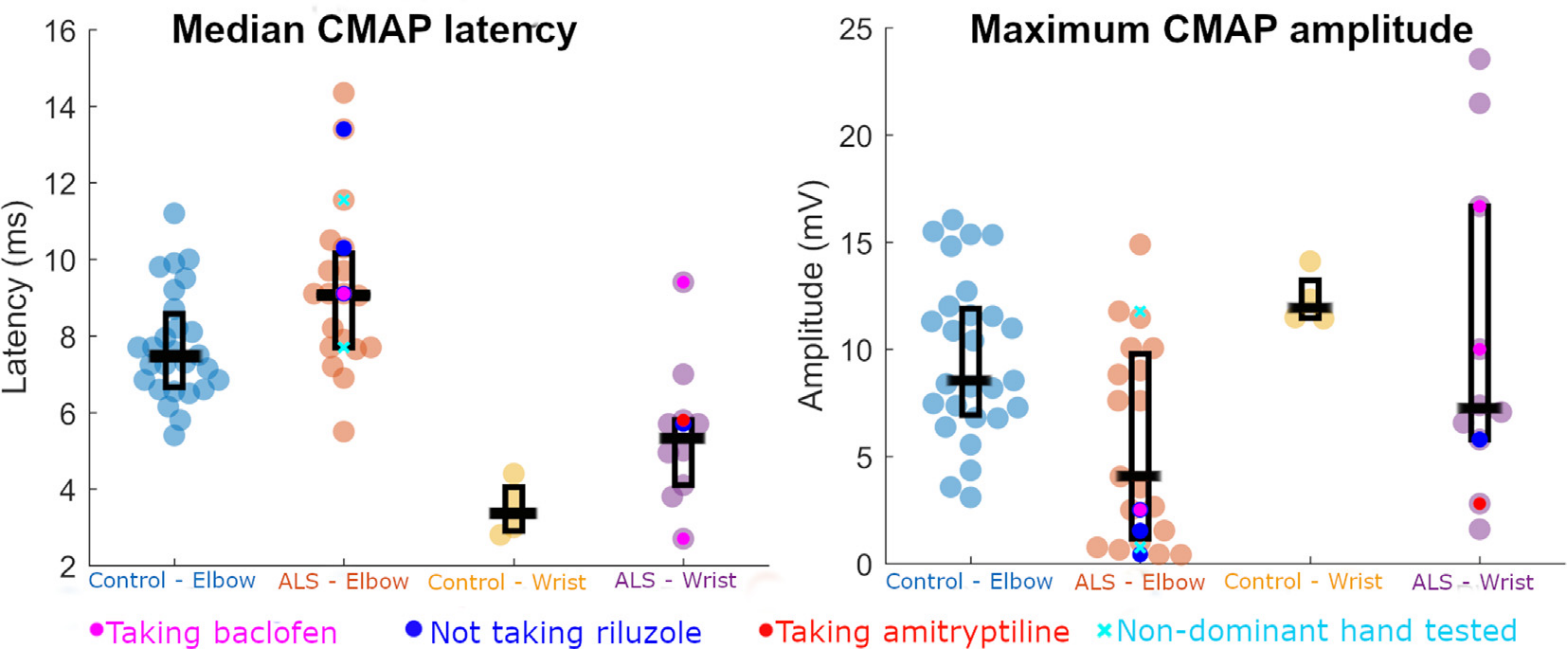
**People with ALS show significantly longer median CMAP latency (left) and smaller maximum CMAP amplitudes (right), compared to controls.** Boxes represent the interquartile range, with horizontal black line within each box representing the median. Cyan crosses represent values of those where the non-dominant hand was tested. Magenta dots represent values of those taking baclofen. Bright red dots represent values of those taking amitriptyline. Bright blue dots represent values of those not taking riluzole. CMAP – Compound muscle action potential. (For interpretation of the references to colour in this figure legend, the reader is referred to the web version of this article.)

Median CMAP latency was longer in those with ALS compared to controls (Fig. 4 left, p = 0.035, d[95 % confidence interval] = 0.28[0.020–0.54]). CMAP amplitude was smaller in those with ALS (Fig. 4 right, p = 0.005; d[95 % confidence interval] = -0.38[-0.64- −0.12]). Group*stimulation site interactions were not statistically significant for amplitude (p = 0.491) or latency (p = 0.565), indicating that groups did not differ in how CMAP values were influenced by the location at which electrical stimulation was applied. Omitting those taking baclofen or amitriptyline or those for whom the non-dominant hand was tested did not affect these findings (values for those in these subcohorts are illustrated in Fig. 4), with the exception of latency not being found to be significantly longer in ALS if only dominant hand measurements were included (p = 0.060). All other p values and effect sizes for the Group coefficient remained similar when analysis was repeated with omission of individuals from any of these subcohorts (latency: p = 0.030–0.035, d = 0.25–0.30, amplitude: p = 0.006–0.009, d = -0.36 - −0.38).

## 4 Discussion

We examined if the use of an AP coil orientation during SICI measurement could provide additional sensitivity in the detection of ALS pathology, compared to the PA orientation. We also examined if we could replicate prior findings of ALS-related changes in SICI and ICF using a novel, fully automated, PEST-based threshold tracking TMS protocol.

### 4.1 Determining the value of anterior-to-posterior coil orientation for capturing ALS pathology

A number of studies in control cohorts have demonstrated that SICI_3ms_ is of greater magnitude when evoked with AP coil orientation compared to the typically-employed PA coil orientation(Cerins et al., 2022, Cirillo et al., 2018, Cirillo and Byblow, 2016, Sale et al., 2016). We therefore hypothesised that motor cortical disinhibition in ALS, measured with SICI_3ms_, would be substantially more evident when measured with AP coil orientation compared to PA coil orientation. First, we replicated previous findings that SICI_3ms-AP_ is of significantly greater magnitude than SICI_3ms-PA_ in controls. However, we did not find disinhibition to be substantially more evident in ALS when employing AP coil orientation. The overlapping 95 % confidence intervals of the AUROC and Hedge’s g values for these two measures indicated similar discrimination abilities.

Notably, the magnitude of SICI_3ms-AP_ and SICI_3ms-PA_ values within individuals did not exhibit a positive correlation in either controls (rho = 0.04) or people with ALS (rho = -0.36). It appears, therefore, that these measures capture distinct facets of motor network activity induced by this paired pulse TMS protocol. A similar lack of correlation between SICI_3ms-PA_ and SICI_3ms-AP_ measures was recently reported in controls (Pavey et al., 2023). The study of Pavey et al., (2023) differs slightly from the control aspect of this study in that serial tracking of SICI measures across 1–7 ms ISIs was employed, as opposed to independent tracking of each measure (as employed here). Serial tracking has been found to bias SICI_3ms_ values towards the values of SICI_1-2.5ms_ (Tankisi et al., 2022), potentially blurring the distinction between these measures. This might explain why Pavey et al., did not find SICI_3ms-AP_ to be of significantly greater magnitude than SICI_3ms-PA_. The study of Pavey et al., provides important additional insights as a broader range of ISIs were examined with AP, PA and lateromedial coil orientations. In keeping with the rationale underpinning the ISIs selected for our protocol, Pavey et al. found that SICI_AP_ and SICI_PA_ were of greatest magnitude when 1 and 3 ms ISIs were employed.

We subsequently averaged values across SICI measures recorded with different coil orientations and ISIs to determine whether the composite would improve the sensitivity with which people with ALS could be discriminated from controls. The AUROC for the composite was not greater than for SICI_3ms-AP_ alone (or markedly different from that of SICI_3ms-PA_ alone). This finding might be explained by ALS causing impairment to a physiological element common to AP and PA SICI_3ms_, orthogonal to the dimension on which these measures are discriminated (i.e., accounting for their relatively low inter-correlation in controls and the overall cohort). However, if ALS were causing such an impairment, that is captured correspondingly by both measures, a positive correlation between the measures would be expected in those with ALS. Instead, we observed a weak negative correlation between the measures in people with ALS (rho = -0.36, p = 0.273). While our correlation analyses including AP-based measures are more limited in statistical power due to fewer available datapoints, even the combined cohort (n = 29) showed no correlation between these measures (rho = 0.19, p = 0.313). Further, those with ALS who exhibited the lowest degree of SICI_3ms-PA_ had relatively high SICI_3ms-AP_ values and vice versa. Therefore, our results suggest that the nature of motor cortical dysfunction, and the TMS measures which best detect it, differs across individuals with ALS. As such, averaging across discordantly affected SICI measures may not produce a measure of improved discriminative power. Overall, our findings indicate that measuring SICI with both AP and PA coil orientations can provide additional measures of motor cortical pathophysiology in ALS, but these measures should be considered separately, and not averaged.

Such discordant effects of ALS on SICI_PA_ and SICI_AP_ within an individual might be explained by differences in the contribution of I-wave facilitation (or short ICF, SICF) to these two measurements. When applying standard stimulation parameters used to evoke SICI, SICF can be concurrently evoked (Awiszus et al., 1999), despite a net inhibitory effect being observed. Therefore, lower SICI_3ms_ measurements may reflect a combination of greater facilitation and/or less inhibition. SICF_PA_ has been shown to be elevated in ALS (van den Bos et al., 2018), such that little/absent SICI_3ms-PA_ in ALS may in part be a reflection of increased I-wave facilitation. However, Pavey et al., (2023) found that, in healthy controls, SICF_3ms_ is evoked only when PA, and not AP, coil orientation is applied. Therefore, enhanced I-wave facilitation might not contribute to a lower SICI_3ms-AP_. If this is the case, individuals with ALS with increased I-wave facilitation would be expected to show lowering of SICI_PA_, not SICI_AP_. By contrast, individuals with relatively unaffected I-wave facilitation but decline in GABA-Aergic activity would be expected to show lowering of SICI_AP_ to a greater extent than SICI_PA_.

However, a number of further studies are warranted to test if this hypothesis explains our observations. For example, pharmacological studies of the relationship between change in tracked SICI measures and GABA-Aergic function (e.g. employing benzodiazepines) remain to be performed in ALS, while epidural recording studies (albeit practically challenging to perform) would enable examination of the contribution of I-wave facilitation to SICI_AP_ and SICI_PA_ measures in ALS. Future TMS-based studies which concurrently examine SICF and SICI with both AP and PA coil orientations in ALS are also required. Finally, we found no correlations between SICI and metrics of disease progression (ALSFRS-R and disease duration) to indicate that disease stage explains differences in extent of SICI_PA_ and SICI_AP_ lowering between individuals with ALS. However, longitudinal examination of these measures is warranted in ALS to more definitively determine the contribution of disease stage to this heterogeneity.

It is important to note that when using AP stimulation, a higher intensity is required to evoke MEPs than when the orientation that induces PA current flow is used. As such, the elicitation of SICI with AP stimulation may not be possible for some people with ALS who exhibit high RMT values due to muscle wasting or cortical inexcitability. However, those at very early symptomatic stages of ALS, when diagnostic markers would be applied clinically, have been reported to have normal, or even reduced, motor thresholds (Huynh et al., 2019), such that SICI_AP_ is likely to be feasibly measured at this early stage.

### 4.2 Replication of previously reported findings in ALS

While lower SICI_1ms-PA_ was observed in people with ALS, this was only observed when specific medications were excluded, or when estimated values for those with thresholds > 100 % MSO were included, indicative of the heterogeneity with which SICI_1ms_ is affected in ALS. Tankisi et al. (Tankisi et al., 2022) and Vucic et al. (Vucic et al., 2011) noted that SICI_3ms-PA_ provides marginally superior discrimination of people with ALS from controls compared to SICI_1ms-PA_. While we also report a greater AUROC for SICI_3ms-PA_ than SICI_1ms-PA_ (0.70 and 0.66 respectively) the AUROC values for the two conditions show broad, considerably overlapping corresponding confidence intervals (see Table 1), indicating that the extent to which SICI_1ms_ and SICI_3ms_ are lowered is heterogenous between individuals with ALS. As these different SICI variants are thought to be associated with discriminable physiological processes(Fisher et al., 2002, Stagg et al., 2011, Ziemann et al., 2015), averaging may mask clinically or prognostically relevant differences in cortical pathophysiology that discriminate among individuals with ALS. Notably, we observed a significant, weak, positive correlation (rho = 0.37, p = 0.006) between SICI_3ms-PA_ and SICI_1ms-PA_ when cohorts were combined. A positive correlation between these measures has previously been identified in older, but not younger, healthy adults (Hermans et al., 2018). Therefore, this relationship may reflect an aspect of the aging process which similarly affects both measures, rather than a common physiological underpinning of both measures. It must be highlighted that the specific physiology reflected by SICI_1ms_ is difficult to interpret, as the literature surrounding its underpinnings remains limited and, in some cases, to be replicated (Fisher et al., 2002, Stagg et al., 2011).

ICF did not differ between people with ALS and controls. Although, when considered at group level the conditioning stimulus had facilitatory effect for the controls, this was not the case for every individual. Moreover, ICF_10ms_ was strongly correlated with SICI_3ms_. This high degree of association and the inconsistent manifestation of ICF_10ms_ among healthy individuals accords with the supposition that the inhibitory networks generating SICI are also activated during ICF(Schwenkreis et al., 1999, Ziemann et al., 1998). This consideration, taken together with indications that ALS-related variations in ICF do not conform to a clear pattern(Geevasinga et al., 2014, Vucic and Kiernan, 2006), suggest that this specific measure is very unlikely to have diagnostic or prognostic utility.

### 4.3 Limitations

As participants in this study were also participating in further research studies on the same day, only specific combinations of ISI and coil orientation were tested to minimise participant fatigue. While averaging between SICI values was performed to provide some comparability to other studies of SICI in ALS, our “average” SICI measures are not directly comparable to those calculated based on a more detailed spectrum of ISIs. Further, AP coil orientation was only tested for SICI_3ms_, and not SICI_1ms_ (see Experimental Paradigm for the rationale of protocol selection). Going forward, further examination of SICI using a wider range of ISIs is warranted using AP coil orientation to determine the extent of additional information on cortical pathophysiology which can be obtained from use of this coil orientation. We did not use a neuronavigation device in this study, instead employing a comprehensive, landmark-based system to ensure consistency of coil positioning. Nonetheless, in the absence of a neuronavigation device, we cannot preclude the possibility that small deviations in coil positioning occurred between different measurements.

Most participants with ALS in this study were concurrently taking riluzole. Riluzole has no significant effects on CMAP, RMT or ICF(Sommer et al., 1999, Stefan et al., 2001), and only have transient effects on SICI (both with 1 and 3 ms ISIs) which return to baseline after 12 weeks(Geevasinga et al., 2016, Stefan et al., 2001). As all of those taking riluzole who were included in this study had been treated for longer than 12 weeks, we do not believe this treatment affects our findings.

Regarding heterogeneity between individuals, a recent study demonstrated that SICI_PA_ normalises in ALS with greater upper motor neurone impairment (Tankisi et al., 2021b). As we did not collect a score specifically of upper motor neurone symptom severity, we cannot investigate how this factor may have contributed to heterogeneity of SICI in people with ALS.

Finally, it is important to highlight that, when using a threshold tracking approach, very good agreement (intraclass correlation of 0.75–0.9) has been shown between intraindividual SICI measurements. While this indicates that these measures can be employed reliably for research, caution is still warranted when applying these measures for clinical decision making purposes (Nielsen et al., 2021).

### 4.4 Conclusion

The use of a coil orientation that induces AP directed current as an alternative to using PA coil orientation, provides, at most, modest improvement in the sensitivity with which an SICI can detect ALS. However, measuring SICI with both AP coil orientation and PA coil orientation provides a more comprehensive characterisation of ALS-associated motor cortical disinhibition, and may permit the discrimination of distinct pathological processes.

## CRediT authorship contribution statement

**Roisin McMackin:** Conceptualization, Funding acquisition, Data curation, Writing – original draft, Writing – review & editing, Investigation, Formal analysis, Methodology, Resources, Project administration, Software. **Yasmine Tadjine:** Investigation, Writing – review & editing. **Antonio Fasano:** Conceptualization, Investigation, Writing – review & editing. **Matthew Mitchell:** Investigation. **Mark Heverin:** Data curation, Writing – review & editing. **Friedemann Awiszus:** Writing – review & editing, Visualization, Investigation, Methodology, Software. **Bahman Nasseroleslami:** Conceptualization, Funding acquisition, Writing – review & editing, Methodology, Supervision. **Richard G. Carson:** Conceptualization, Writing – review & editing, Methodology, Project administration, Supervision. **Orla Hardiman:** Resources, Funding acquisition, Conceptualization, Writing – review & editing, Supervision.

## Figures and Tables

**Supplementary figure 1:**
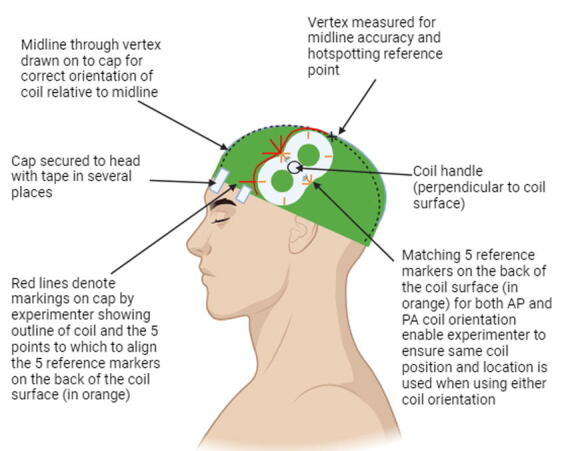


**Supplementary figure 2:**