# Supplementary material for: Examining short interval intracortical inhibition with different transcranial magnetic stimulation-induced current directions in ALS
Source: Clin Neurophysiol Pract. 2024 Mar 13;9:120–9. doi: 10.1016/j.cnp.2024.03.001 (PMC11002888; doi:10.1016/j.cnp.2024.03.001)
Supplement: Supplementary data 1 [file mmc1.docx]

**SUPPLEMENTARY MATERIAL**

**Supplementary Methods**

Included medications

An exception was made to include those taking baclofen (GABA_B_ agonist(de Beaurepaire, 2018)) or amitriptyline (inhibitor of serotonin and noradrenalin transporters(Lawson, 2017)) due to the prevalence of their use in those with ALS, to allow for increased recruitment. A 50mg/day dose of baclofen has been reported to reduce ICF(Ziemann et al., 1996a) (2-5hrs) post-dosing. This dose of baclofen has also been reported both to increase(Ziemann et al., 1996a) and reduce SICI_3ms_(McDonnell et al., 2006) acutely (1.5-5hrs post-dosing). However, these effects on ICF and SICI have been reported to subside by 24hrs post-dosing(Ziemann et al., 1996a), and chronic baclofen use (>1 year, >60mg/day) has been found not to affect SICI_2.5ms_(Barry et al., 2013). All these studies found that baclofen did not affect RMT. The effects of amitriptyline itself on these TMS-based measures has not, to our knowledge, been tested, however selective serotonin or noradrenalin reuptake inhibitors (SSRIs/SNRIs), which also enhance central serotonergic or noradrenergic activity respectively, have. Studies of SSRIs have repeatedly found no effect on SICI (across 1-5ms ISIs) or RMT, and conflicting effects of increase and decrease in ICF have been described(Caipa et al., 2018; Gerdelat-Mas et al., 2005; Ilic et al., 2002; Pleger et al., 2004). However, SNRIs have been reported to reduce SICI_2-3ms_ and increase ICF, with no effect on RMT(Chen et al., 2014; Kuo et al., 2017). Therefore use of these medications has been accounted for in our analysis.

As riluzole was being taken by to most individuals diagnosed with ALS, and riluzole has been found to have no significant effects on CMAP, RMT or ICF(Sommer et al., 1999; Stefan et al., 2001), and to only have transient effects on SICI (both with 1 and 3ms ISIs) which return to baseline after 12 weeks(Geevasinga et al., 2016; Stefan et al., 2001), those taking riluzole were included. All participants who were taking riluzole and who could contribute paired pulse TMS measures were taking riluzole for more than 12 weeks at the time of recording.

TMS hotspotting

Stimulator output was gradually increased from ~30% of maximum stimulator output (% MSO) in 10% MSO increments 2cm anterior and 5cm lateral from the vertex, until an MEP was elicited in the target muscle, or until 70% MSO was reached. Thereafter, the coil was moved in ~1 cm increments along either the anterior-to-posterior or medial-to-lateral axis from this point and the stimulus intensity increased or reduced until the position which elicited an MEP at the lowest stimulation intensity was identified.

*Coil positioning protocol*

A fitted cloth cap was put on the participant’s head and secured to the head. The position of the coil at the identified hotspot (including coil location on the head and angle relative to the midline of the head) was clearly marked on the cloth cap by drawing an outline of the coil and markings which aligned to 5 landmark points located on the back of the coil’s surface (Figure S1). The coil used to deliver these pulses is symmetrical (DeyMed 50BFT figure-of-eight coil) and has 5 matching AP landmark points located on the back of the coil’s surface facing the opposite direction. As such, when the coil is rotated by 180 degrees, achieving AP positioning in the exact same location, the markings drawn previously on the cap will align exactly with these AP landmark points and the coil’s outline. Therefore, we can be highly confident that the same position on the head was targeted whether AP or PA coil orientation was used.

Electromyography recording protocol

Bipolar electromyographic (EMG) activity was recorded from the abductor pollicis brevis (APB) using pairs of Ag-AgCl electrodes (Cleartrace 1700, ConMed, Haverhill, MA, USA) spaced approximately 2cm apart in a belly-tendon montage. The APB was recorded from the dominant hand (as determined by the Edinburgh Handedness Index(Oldfield, 1971)). An exception was made if single pulse threshold values could only be recorded from the non-dominant hand. This was the case for a small number of individuals with ALS. A reference electrode was placed on the ulnar head of the right wrist. EMG signals were amplified (gain = 1000) and band-pass filtered (10–1000 Hz) via BioPac EMG100C amplifiers (Biopac Systems UK, Pershore, UK), digitized at a sampling rate of 10 kHz (Micro1401, CED, Cambridge, UK), and recorded with Signal software (Signal 7.01, CED, Cambridge, UK). Ambient electrical mains-associated noise was subtracted after signal amplification, before digitisation, via a Humbug Noise Eliminator or equivalent D400 Multichannel Noise Eliminator (Digitimer Ltd., Welwyn Garden City, UK).

Adaptive threshold tracking

An adaptive threshold tracking protocol was applied to obtain all single-pulse (i.e., RMT and THT) and paired pulse measures, using maximum likelihood protocol PEST. This procedure utilises a sigmoid-shaped logistic function to determine the stimulation intensity at which there exists a 50% probability of eliciting an MEP with peak-to-peak amplitude greater than a defined threshold (i.e. 50µV for RMT, 200µV for THT and all paired pulse protocol). This function, and its implementation in the commonly used MTAT 2.0 manual interface programme, were described and developed by Prof. Friedemann Awiszus(Awiszus, 2003). Maximum likelihood PEST has previously been used to perform threshold tracking(Cirillo and Byblow, 2016; Mooney et al., 2018), however, here and in our previous publication(Calvert et al., 2020), we have fully automated the procedure using Signal (CED Ltd., Cambridge UK) and MATLAB (R2016a, MathWorks Inc., MA, USA) scripts to reduce probability of human error (for example by misreading response amplitudes or incorrectly setting the recommended stimulation intensity), to reduce required experimenter presence, and to facilitate automated baseline amplitude screening and trial rejection. Briefly, following each pulse, peak-to-peak MEP amplitude is calculated immediately following pulse-associated EMG digitisation. Peak-to-peak amplitude of the MEP is then passed to a PEST algorithm to determine the next stimulation intensity, to which the stimulator is then set automatically in advance of next pulse delivery, until the estimated threshold is deemed within the safety guidelines of Rossi et al.(Rossi et al., 2009) (i.e., estimated threshold within 95%-105% of true threshold). To achieve this level of confidence, we required 20 single/paired pulse “trials” to be input to PEST if no a priori information is provided regarding the range of stimulation intensities within which the threshold will fall. This number of trials is based on the exact calculation of the variance by complete enumeration. In this case, a large range of stimulus intensities (ranging from 15-100% MSO) is considered. For some later participants (with addition of new features to our automated PEST codes), trial number could be reduced to 18 by predefining a narrower upper and lower limit of stimulation intensities to search. Such limits were defined where a specific stimulation intensity consistently did not evoke MEPs (set as the lower limit) and 1.5 times this value consistently did evoke MEPs above threshold (set as the upper limit), such as 54% and 81% MSO.

In addition to calculation of MEP amplitude following each stimulus, root mean square amplitude in the baseline window immediately preceding first stimulus onset (i.e., the conditioning stimulus in the case of paired pulse protocol, the test stimulus onset in the case of single pulse protocol) was also calculated, and where this amplitude was >10µV, the MEP was not passed to the PEST algorithm and the pulse trial was repeated. The experimenter also visually monitored the EMG during data collection to identify where lower motor neuron-associated EMG abnormalities (e.g., fasciculations, fibrillations) occurred within the MEP-peak search window and not in the baseline, which could mislead the PEST algorithm. In such cases the protocol was terminated and restarted. Following collection of 4 ALS and 9 control datasets, the baseline window was expanded from 50ms to 200ms to improve automated detection of these artefacts, but experimenter monitoring of signal input was maintained.

*Compound muscle action potential measurement*

A bar electrode containing two steel contacts (0.8cm diameter) holding saline-soaked felt pads with a fixed distance of 3cm between the cathode and anode was used to deliver stimuli of 100µs width. Stimulation was initiated at 10mA and increased in 10mA increments until CMAP peak-to-peak amplitude no longer increased, followed by increase in stimulus amplitude by 10-20% to ensure supramaximal threshold stimulation. The maximal possible stimulus intensity was 99.99mA (device limit). Participant comfort was continuously monitored between stimuli. Participation in the electrical nerve stimulation phase of the protocol was entirely voluntary.

*Compound muscle action potential analysis*

CMAP trials with a 200ms pre-stimulus baseline root mean square amplitude >25µV were rejected. Onset thresholds were defined for each CMAP as the mean baseline EMG amplitude plus/minus 20μV. Latency of each CMAP was defined as the first time point at which signal amplitude at this time and the 14 following data points crossed one of these onset thresholds, with more than half of each of these 15 datapoints being farther from baseline than the preceding datapoint. Median CMAP latency was determined across trials. Maximum CMAP amplitude (mCMAP) was determined as the maximum peak-to-peak amplitude across CMAPs recorded for each individual. Among early participants (4 ALS, 8 controls) when 1000 gain was used, amplifier saturation was identified by the tips of the positive/negative CMAP peak flat-lining at +/- 5V respectively. In such cases, peaks were repaired by spline interpolation prior to maximum CMAP peak-to-peak amplitude calculation. The accuracy of peak-to-peak amplitude calculation following this repair method was validated using 15 CMAPs where 500 gain was applied and the positive or negative peak was greater than 5mV (i.e., values which would have been lost due to amplifier saturation were gain set to 1000). In these data, peak amplitude values above 5mV were artificially removed and recalculated by spline interpolation, with the resulting peak-to-peak amplitude being compared to that calculated from the true signal. Lin’s concordance correlation coefficient was 0.997, demonstrating almost perfect agreement (>0.99)(Akoglu, 2018) between real signal and interpolation-based signal CMAP amplitude measurements. Following identification of this issue, amplifier gain was reduced to 500 during CMAP recording to avoid amplifier saturation (at +/- 5V), whereafter amplifier saturation occurred for only one control and one participant with ALS (for whom peaks were repaired by spline interpolation). Reduction in amplifier gain was accounted for by the multiplication of all CMAP signal amplitudes by two in these individuals.

**Supplementary Results**

*Data collection limitations*

One control and four people with ALS were excluded upon attendance of the study session due to inability to relax and provide consistently low baseline amplitude EMG, while six people with ALS (but no controls) had to be excluded due to inexcitability or very high thresholds resulting in inability to record RMT_PA_. Of the 35 people with ALS from whom RMT_PA_ was recorded, a further 8 people with ALS had THT values >100% MSO/could not generate the MEPs required to record THT. These 14 people (5 female, age median [range]: 62.25 [43-76] years) had a median time since diagnosis of 5 months (range:1-24 months), with three experiencing bulbar onset and 11 experiencing spinal onset. Total ALSFRS-R scores were available for 10 of these people, with a median score of 38.1 (range: 30-45). The subcohort of 27 people with ALS who provided paired pulse TMS data (8 female, age median [range]: 64.33 [41.25-78.75] years) were similarly sex- (chi^2^=0.0099, p=0.921) but not age-matched (p=0.041) to the control cohort. Three people with ALS were taking baclofen, two of whom provided paired pulse data. Four people with ALS were not taking riluzole, two of whom provided paired pulse data. One person, who provided paired pulse data, was taking amitriptyline. No participants were taking both baclofen and amitriptyline, although one person taking baclofen was not taking riluzole (for over 12 weeks). The non-dominant (left) hand APB was recorded in 4 people with ALS as RMT_PA_/THT_PA_ could not be recorded from the dominant (right) hand but could be obtained from the non-dominant side. Those in whom recordings were performed on the non-dominant hand were all taking riluzole and not taking other neuroactive medications. Some participants from whom RMT and THT were recorded could not provide all the remaining measurements due to high 200µV threshold values resulting in thresholds >100% MSO in the presence of inhibitory conditioning stimuli.

**Figure S1. Illustration of the procedure used to ensure consistent coil positioning for delivery of test pulses using both anterior-to-posterior (AP) and posterior-to-anterior (PA) coil orientations.** Figure created using BioRender.

**Figure S2. Lower SICI in ALS remains present if estimated values for those with conditioned thresholds >100% MSO are included.** Boxes represent the interquartile range, with black horizontal line within each box representing the median. Cyan crosses represent values of those where the non-dominant hand was tested. Magenta dots represent values of those taking baclofen. Bright red dots represent values of those taking amitriptyline. Bright blue dots represent values of those not taking riluzole. P values in subfigure titles are those for Mann-Whitney U test comparisons between cohorts. SICI – Short-interval intracortical inhibition

**Table S1. Summary statistics for each TMS parameter recorded when values estimated for those with conditioned thresholds >100% are estimated.** Numbers of ALS and control datasets recorded are listed under Cn and Pn respectively. Numbers of control and ALS datasets still excluded due to CTT>100% MSO (as 50µV MEPs were not evoked at 100%MSO in the presence of the conditioning stimulus) are listed under C>100 and P>100 respectively. Group p (g) column values refer to p-value and Hedge’s g for the comparison of ALS and control values. 95% CI – 95% confidence interval. SICI – Short-interval intracortical inhibition. ISI – Interstimulus interval. PA – Posterior-to-anterior. AP – Anterior-to-posterior. P values listed are uncorrected. Those coefficient values with corrected p values (at 5% false discovery rate) < 0.05 are emboldened. AUROC – Area under the receiver operating characteristic curve.

| Parameter | Orientation | ISI | Cn | C  >100 | Pn | P  >100 | Group p (g [95%CI]) | AUROC  (95% CI) |
| --- | --- | --- | --- | --- | --- | --- | --- | --- |
| SICI | PA | 1ms | 39 | 0 | 26 | 0 | **0.016 (-0.42**  **[-1.02-0.06])** | **0.63**  **(0.49-0.77)** |
|  |  | 3ms | 38 | 1 | 27 | 0 | **0.002 (-0.58**  **[-1.22- -0.09])** | **0.70**  **(0.56-0.81)** |
|  |  | Mean | 39 | N/A | 24 | N/A | **<0.001 (-0.99**  **[-1.52- -0.48])** | **0.77**  **(0.63-0.89)** |
|  | AP | 3ms | 27 | 3 | 19 | 1 | **0.004 (-0.75**  **[-1.50- -0.19])** | **0.76**  **(0.60-0.90)** |
|  | AP and PA | 3ms | 27 | N/A | 19 | N/A | **<0.001 (-1.06**  **[-1.85 - -0.50])** | **0.80**  **(0.62-0.91)** |
|  |  | Mean | 27 | N/A | 18 | N/A | **0.005 (-1.01**  **[-1.70- -0.41])** | **0.75**  **(0.56-0.89)** |
